# Association of Sarcopenia with Pain and Disability in Hand Osteoarthritis: A Retrospective, Cross-Sectional, Single-Center Study

**DOI:** 10.5152/ArchRheumatol.2025.25088

**Published:** 2025-12-01

**Authors:** Hanna Lee, Sang-Il Lee, Yun-Hong Cheon, Hyunjin Lim, Ki Soo Park, Hyun-Ok Kim

**Affiliations:** 1 Division of Rheumatology, Department of Internal Medicine, Gyeongsang National University Hospital, changwon, South Korea; 2 Department of Medicine, Gyeongsang National University, Jinju, South Korea; 3 Division of Rheumatology, Department of Internal Medicine, Gyeongsang National University Hospital, Jinju, South Korea; 4 Department of Preventive Medicine, Gyeongsang National University College of Medicine Institute of Health Sciences, Jinju, Korea

**Keywords:** Bioelectrical impedance analysis, functional disability, hand osteoarthritis, muscle mass, sarcopenia

## Abstract

**Background/Aims:**

Sarcopenia is known to worsen clinical outcomes in osteoarthritis, particularly in weight-bearing joints, yet its relationship with symptom burden in hand osteoarthritis has not been well established. This study explored the relationship between sarcopenia, hand pain, and functional status in patients with hand osteoarthritis.

**Materials and Methods:**

A retrospective analysis was conducted on 1139 patients aged ≥40 years with radiographically confirmed hand osteoarthritis. Sarcopenia was defined as the ratio of muscle mass to body mass index, measured via bioelectrical impedance analysis. Hand pain and function were assessed using the Australian/Canadian Osteoarthritis Hand Index, and the Disabilities of the Arm, Shoulder and Hand (DASH) questionnaires. Sex-specific multivariable linear regression models were constructed, adjusting for demographic and lifestyle covariates.

**Results:**

In males, lower appendicular skeletal muscle mass/body mass index was significantly associated with higher DASH scores (estimate: −10.664, *P *= .006). A significant association with higher Australian/Canadian Osteoarthritis Hand Index scores was also observed (estimate: −26.236, *P* = .030). Lower upper-extremity muscle mass/body mass index was likewise associated with higher DASH scores in males (estimate: −41.074, *P*= .013). In females, none of the associations reached statistical significance.

**Conclusion:**

These findings suggest that sarcopenia contributes to increased pain and disability in hand osteoarthritis, highlighting the clinical importance of preserving muscle mass in its management.

**Main Points**

- In men with hand osteoarthritis (OA), reduced upper-extremity muscle mass and lower appendicular skeletal muscle/body mass index (ASM/BMI) were significantly associated with higher Disabilities of the Arm, Shoulder and Hand scores, reflecting impaired function.
- Associations between muscle mass and Australian/Canadian Osteoarthritis Hand Index (AUSCAN) pain scores showed negative trends in men but did not consistently reach statistical significance.
- In women, neither upper-extremity muscle mass nor ASM/BMI demonstrated significant associations with AUSCAN or DASH outcomes.
- Despite modest explanatory power (low *R*² values), muscle mass emerged as an independent determinant of upper-extremity function in men.
- These findings suggest that muscle preservation may serve as a protective factor in hand OA, potentially mitigating pain and functional decline through mechanical and inflammatory pathways.

## Introduction

Osteoarthritis (OA) is a common degenerative joint disease affecting multiple joints, particularly the knees and hands, and is a leading cause of pain, functional disability, and reduced quality of life in older adults.^1^^,^^2^ The hands are frequently used in daily activities, and degenerative changes accompanied by pain in these joints can significantly impair functional performance.^2^^,^^3^ To assess pain and functional limitations in hand OA, validated instruments such as the Australian/Canadian Osteoarthritis Hand Index (AUSCAN) and the Disabilities of the Arm, Shoulder and Hand (DASH) questionnaire are commonly used.^4^^,^^5^ Although several factors—advanced age, increased body mass index (BMI), lower socioeconomic status, comorbid rheumatoid disease, and decreased grip strength—have been linked to worse hand OA symptoms, evidence remains limited.^6^ ^7^

Knee OA is commonly assessed with the Western Ontario and McMaster Universities Osteoarthritis Index (WOMAC). Higher WOMAC scores are associated with older age, elevated BMI, reduced cartilage thickness, and structural changes such as osteophyte formation.^7^ ^8^ In patients with knee OA, decreased muscle mass—particularly of the quadriceps—has been linked to greater pain and diminished functional performance, highlighting the protective role of muscle mass in joint stabilization and load modulation.^9^ ^10^ Declining muscle mass has also emerged as a major determinant of functional limitation and a growing concern in geriatric medicine.^11^

In contrast, the relationship between muscle mass and clinical outcomes in hand OA remains underexplored. Because reduced muscle mass can influence pain perception and physical capacity, it may similarly contribute to increased pain and functional impairment in hand OA.^12^ Identifying factors such as muscle mass that influence pain and function is essential for predicting disease progression and implementing strategies to prevent further deterioration.

Therefore, this study aimed to examine whether sarcopenia is associated with increased pain and poorer functional outcomes in patients with hand OA. Clarifying the role of muscle mass in the clinical course of hand OA may enhance understanding of its pathophysiology and support the development of therapies aimed at muscle preservation.

## Methods

### Study Design and Participants

We performed a retrospective, cross-sectional study at Changwon Gyeongsang National University Hospital, enrolling patients aged 40 years or older who had radiographically verified hand OA between January 2018 and December 2020. Diagnosis was based on the Kellgren–Lawrence (KL) grading system. Individuals with inflammatory arthritis (e.g., rheumatoid arthritis) or prior upper-limb trauma, or a history of hand surgery, were excluded. Ethics Committee of University of Gyeongsang National University Changwon Hospital (Approval no: 2025-07-001-003, Date: August 6, 2025). Due to the retrospective nature of the study, informed consent was waived.

**Figure 1.**
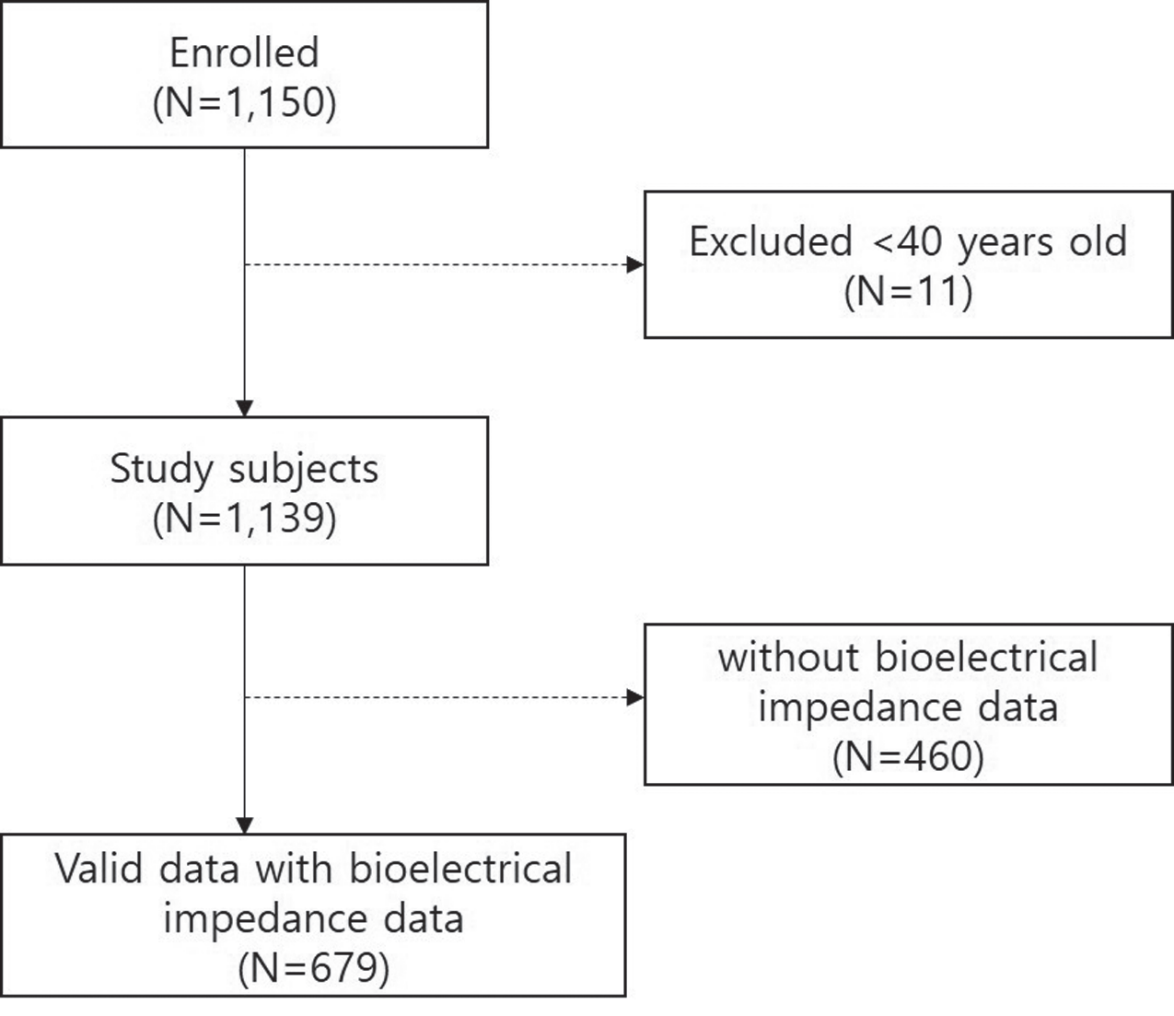
Flow diagram of study participants. A total of 1150 individuals were initially enrolled. After excluding 11 participants younger than 40 years, 1139 subjects remained eligible. Among these, 460 without available InBody data were excluded. Finally, 679 participants with valid InBody measurements were included in the final analysis.

A total of 1150 individuals were enrolled. After excluding 11 participants younger than 40 years and 460 without bioelectrical impedance data, 679 participants who completed bioelectrical impedance measurements were included in the analysis (Figure 1).

Demographic and clinical data collected included sex, age, educational level (≤ elementary, middle, or ≥ high school), smoking status (never, former, or current), alcohol consumption (never, or current), hypertension, diabetes, and radiographic OA severity (KL grade ≥3 in multiple joints).^13^ Body mass index was calculated as weight/height² (kg/m²). This study was approved by the Institutional Review Board of xxx (Approval No.: 2025-07-001-003; Date: August 06, 2025).

### Assessment of Muscle Mass and Sarcopenia Definition

Muscle mass was evaluated using a multifrequency bioelectrical impedance analyzer (InBody, Biospace Co., Seoul, Korea).^14^ Sarcopenia was defined according to the Foundation for the National Institutes of Health sarcopenia criteria, with low muscle mass identified using the ratio of appendicular skeletal muscle mass to BMI (ASM/BMI), with cutoff values of <0.789 for men and <0.512 for women.^15^ The index was calculated as follows:



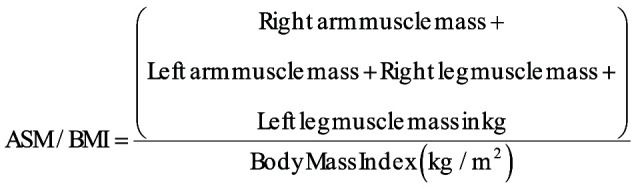





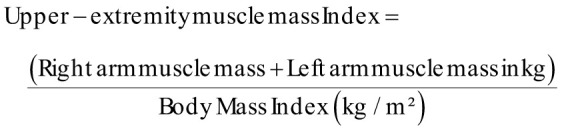



### Assessment of Pain and Function

Hand function and pain were evaluated using the DASH questionnaire and AUSCAN, respectively.^4^^,^^5^ The DASH score ranges between 0 and 100, with higher scores indicating greater upper-extremity disability. The AUSCAN index ranges between 0 and 300, with higher scores indicating worsened symptoms. Both assessments were performed concurrently with radiographic evaluations for each participant.

### Statistical Analysis

Descriptive statistical methods were applied to present the baseline characteristics of the study cohort. Continuous variables are reported either as mean values with SDs or as median values accompanied by interquartile ranges, and categorical variables are presented as counts and proportions.

Multivariate logistic and linear regression analyses were conducted separately for male and female participants to examine the association between sarcopenia and key outcomes, including hand pain and functional impairment. Models were adjusted for potential confounders such as age, BMI, educational level, smoking status, alcohol consumption, hypertension, diabetes mellitus, and radiographic severity. Model performance was assessed using *R*² and adjusted *R*² values.

### Handling of Missing Data

To manage missing values, a multiple imputation approach with chained equations was employed under the assumption of data being missing at random. Twenty imputed datasets were created, and the final estimates were derived by pooling the results using Rubin’s rules.

All statistical analyses were performed using R software version 4.3.1 (R Core Team; Vienna, Austria), with a *P*-value of <.05 considered statistically significant.

## Results

### Baseline Patient Characteristics

Table 1 summarizes the baseline characteristics of the study population. A total of 1139 patients with hand OA were included. The mean age was 60.0 ± 8.3 years, and 584 (51.3%) were female. Educational attainment was distributed as follows: ≥ high school (49.1%), ≤ elementary school (30.2%), and middle school (20.7%). Current smokers comprised 12.5% of the cohort, while 74.0% reported alcohol consumption. The prevalence of hypertension and diabetes mellitus was 29.6% and 11.0%, respectively. Obesity, defined as a BMI ≥ 25 kg/m², was observed in 43.7% of the cohort. Moderate-to-severe hand OA was identified in 19.1% of participants.

### Association of Variables with Hand Function and Pain

In males, with the AUSCAN score as the dependent variable, the ratio of upper-extremity muscle mass to BMI was negatively associated with the AUSCAN score, but this was not statistically significant (estimate = −70.199, *P* = .115). In females, the ratio of upper-extremity muscle mass to BMI was not significantly associated with the AUSCAN score (estimate = 65.259, *P* = .476). Age, education level, and smoking status were not significantly associated with AUSCAN scores. Model *R*² values were 0.026 for males and 0.022 for females (Table 2).

### Association of Variables with Upper-Extremity Function

In males, with the DASH score, upper-extremity muscle mass was negatively associated with the DASH score (estimate = −30.248, *P* = .078), but this did not meet the threshold for statistical significance. In females, upper-extremity muscle mass was negatively associated with the DASH score (estimate = −19.537, *P  = *.557). Higher educational level showed an inverse association with DASH scores; however, this was not statistically significant. Age and former smoking status were not significantly associated with the DASH score. Model fit was low (male *R*² = 0.035; female *R*² = 0.024; Table 3).

### Relationship Between Muscle Mass and Australian/Canadian Osteoarthritis Hand Index Score

In males, greater upper-extremity muscle mass was associated with lower AUSCAN scores, although the association did not reach statistical significance (estimate = −79.017, *P* = .061). The explanatory power of the model was modest (*R*² = 0.008). In females, no significant association was observed between upper-extremity muscle mass and AUSCAN score (estimate = 45.297, *P* = .591), and explanatory power was negligible (*R^2^* = 0.002).

Similarly, when using appendicular skeletal muscle mass, higher values were significantly associated with lower AUSCAN score in males (estimate = −26.236, *P* = .030), with an explanatory power of *R^2^* = 0.015. In females, however, the relationship between appendicular skeletal muscle mass and AUSCAN score was not statistically significant (estimate = −3.428, *P* = .866), and the explanatory power remained minimal (*R^2^* = 0.001; Table 4).

### Relationship Between Muscle Mass and the Disabilities of the Arm, Shoulder, and Hand Score

In males, greater upper-extremity muscle mass was significantly associated with improved lower DASH scores (better upper-extremity function) (estimate = −41.074, *P* = .013, *R^2^* = 0.015). In females, upper-extremity muscle mass was not significantly associated with the DASH score (estimate = −31.594, *P* = .324, *R^2^* = 0.003)

In males, higher ASM/BMI was significantly associated with lower DASH scores (estimate = −10.664, *P* = .006, *R^2^* = 0.016). In females, a similar negative trend was observed (estimate = −8.600, *P*= .211), but the association was not significant; model fit remained minimal (*R^2^* = 0.004) (Table 5).

## Discussion

In this study of patients with hand OA, lower muscle mass relative to BMI was associated with higher pain and poorer functional outcomes measured by the AUSCAN and DASH scores, but only in males. These findings align with previous research on knee OA, in which reduced muscle mass and strength are correlated with increased pain and functional limitations.^9^ ^10^ Although explanatory power was limited, muscle mass should still be considered an independent determinant of upper-extremity function. Similar to the impact of quadriceps muscle loss on knee joint stability, reduced upper-extremity muscle mass may compromise the stability of the shoulder, elbow, wrist, and hand, thereby increasing mechanical stress on these structures.^16^ This heightened stress may cause microdamage and provoke local inflammation. Moreover, sarcopenia has been linked to systemic low-grade inflammation; for example, a 2024 study suggested that reduced serum β2-microglobulin has been suggested as a candidate biomarker for sarcopenia in older adults, reflecting systemic alterations in muscle and inflammatory homeostasis.^17^ Such systemic inflammation can sensitize peripheral nociceptors and exacerbate pain.^18^^-^21 The resulting worsening of pain and diminished sensory function may, in turn, reduce physical activity, ultimately impairing upper-limb functional performance. Importantly, muscle loss is a modifiable factor and may provide an opportunity for management intervention in hand OA.

One notable limitation of this study is the absence of grip-strength measurement, a key determinant of upper-limb function and a major criterion in the diagnosis of sarcopenia. This limitation arose from reliance on bioelectrical impedance analysis, a practical and accessible method in clinical settings. Despite this limitation, the study is informative in demonstrating a linear relationship between reduced muscle mass and decreased upper-limb function. Further retrospective studies are warranted to explore this correlation in more detail. Future research should focus on patients with hand OA, incorporating interventions such as exercise and nutritional supplementation, along with assessment of muscle mass, upper-limb function, and grip strength.

These results suggest that lower muscle mass contributes to increased hand pain and functional limitations of the upper extremities in hand OA. These findings suggest that sarcopenia may play a key role in the clinical course of hand OA, potentially contributing through both mechanical dysfunction and systemic inflammatory pathways. Considering the rising prevalence of sarcopenia in aging populations, these findings emphasize the need for comprehensive management strategies that incorporate muscle mass preservation to potentially mitigate the burden of hand OA. Prospective studies employing objective assessments of muscle strength and longitudinal follow-up are warranted to elucidate these associations and inform targeted interventions.

## Figures and Tables

**Table 1.** Baseline Characteristics of the Study Population (n = 1139)

**Variables ^a^**	**Total (n = 1139 )**
Sex, n (%)	
Female	584 (51.3)
Male	555 (48.7)
Age (years), mean ± SD	60.0 ± 8.3
40-64, n (%)	788 (69.2)
≥65, n (%)	351 (30.8)
Education, n (%)	
≤Elementary school	344 (30.2)
Middle school	236 (20.7)
≥High school	559 (49.1)
Smoking status, n (%)	
Never	761 (66.8)
Ex-smoker	236 (20.7)
Current smoker	142 (12.5)
Drinking status (yes), n (%)	843 (74.0)
HTN, n (%)	337 (29.6)
DM, n (%)	125 (11.0)
BMI, n (%)	
≥25	498 (43.7)
<25	641 (56.3)
OA severity, n (%)	
Mild	922 (80.9)
Moderate to severe	217 (19.1)

BMI, body mass index; DM, diabetes mellitus; HTN, hypertension; OA, osteoarthritis.

^a^Values are presented in numbers and percentages unless stated otherwise.

**Table 2.** Multivariate Linear Regression Analysis of Factors Associated with the Australian/Canadian Osteoarthritis Hand Index

**AUSCAN Score**	**Estimate**	**CI_lower**	**CI_upper**	***P***
**(a) Association of variable with AUSCAN score in males**				
Upper muscle mass	−70.199	−157.85	17.457	.115
Age (ref. <65)				
Age ≥ 65	−2.229	−7.846	3.388	.433
Education (ref. <elementary school)				
Middle school	0.165	−8.150	8.481	.969
>High school	−6.775	−14.226	0.675	.074
Smoke (ref. never)				
Ex-smoker	1.458	−3.945	6.861	.596
Current smoker	3.514	−2.688	9.716	.266
*R* ^2^	0.026 (0.022-0.032)			
**(b) Association of variable with AUSCAN score in females**				
Upper muscle mass	65.259	−123.16	253.678	.476
Age (ref. <65)				
Age ≥65	4.511	−3.034	12.055	.240
Education (ref. <elementary school)				
Middle school	1.838	−7.319	10.995	.691
≥High school	−6.271	−13.619	1.078	.094
Smoke (ref. never)				
Ex-smoker	18.906	−10.006	47.818	.199
Current smoker	12.661	−18.250	43.573	.421
*R*²	0.022 (0.016-0.025)			

*P*-values were calculated using univariate logistic regression analysis. Boldface indicates statistical significance (*P* < .05).

AUSCAN, the Australian/Canadian Osteoarthritis Hand Index., ref., reference

**Table 3.** Multivariate Linear Regression Analysis of Factors Associated with the Disabilities of the Arm, Shoulder, and Hand

**DASH Score**	**Estimate**	**CI_lower**	**CI_upper**	***P***
**(a) Association of variable with DASH score in males**				
Upper muscle mass	−30.240	−63.96	3.484	.078
Age (ref. <65)				
Age ≥65	1.272	−0.645	3.189	.193
Education (ref. <elementary school)				
Middle school	0.023	−2.733	2.779	.987
≥High school	−2.422	−4.788	−0.056	.045
Smoke (ref. never)				
Ex-smoker	−0.059	−2.085	1.967	.954
Current smoker	1.007	−1.331	3.344	.398
*R*²	0.035 (0.031-0.038)			
**(b) Association of variable with DASH score in females**				
Upper muscle mass	−19.537	−86.50	47.431	.557
Age (ref. <65)				
Age ≥65	1.917	−1.061	4.895	.207
Education (ref. <elementary school)				
Middle school	−1.303	−4.656	2.050	.446
≥High school	−3.500	−6.319	−0.682	.015
Smoke (ref. never)				
Ex-smoker	4.499	−7.110	16.109	.447
Current smoker	3.810	−8.910	16.529	.557
*R*²	0.024 (0.022-0.025)			

*P*-values were calculated using univariate logistic regression analysis. Boldface indicates statistical significance (*P* < .05).

DASH, the Disabilities of the Arm, Shoulder, and Hand., ref., reference

**Table 4.** Relationship Between Muscle Mass and the Australian/Canadian Osteoarthritis Hand Index Score

**AUSCAN**	**Estimate**	**CI_lower**	**CI_upper**	***P***
**(a) Relationship between upper extremity-muscle mass and AUSCAN score in males**				
Upper-extremity muscle mass	−79.017	−161.68	3.648	.061
*R*²	0.008 (0.005-0.013)			
**(b) Relationship between upper-extremity muscle mass and AUSCAN score in females**				
Upper-extremity muscle mass	45.297	−126.34	246.936	.591
*R*²	0.002 (0.000-0.005)			
**(c) Relationship between appendicular skeletal muscle mass and AUSCAN score in males**				
Appendicular skeletal muscle mass	−26.236	−49.78	−2.693s	.03
*R*²	0.015 (0.007-0.023)			
**(d) Relationship between appendicular skeletal muscle mass and AUSCAN score in females**				
Appendicular skeletal muscle mass	−3.428	−45.73	38.872	.866
*R*²	0.001 (0.001-0.003)			

AUSCAN, the Australian/Canadian Osteoarthritis Hand Index.

**Table 5.** Relationship Between Muscle Mass and the Disabilities of the Arm, Shoulder, and Hand Score

**DASH Score**	**Estimate**	**CI_lower**	**CI_upper**	***P***
**(a) Relationship between upper-extremity muscle mass and DASH score in males**				
Upper-extremity muscle mass	−41.074	−73.24	−8.906	.013
*R*²	0.015 (0.009-0.019)			
**(b) Relationship between upper-extremity muscle mass and DASH score in females**				
Upper-extremity muscle mass	−31.594	−95.41	32.222	.324
*R*²	0.003 (0.000-0.005)			
**(c) Relationship between appendicular skeletal muscle mass and DASH score in males**				
Appendicular skeletal muscle mass	−10.664	−18.23	−3.093	.006
*R*²	0.016 (0.012-0.021)			
**(d) Relationship between appendicular skeletal muscle mass and DASH score in females**				
Appendicular skeletal muscle mass	−8.600	−22.20	5.004	.211
*R*²	0.004 (0.001-0.007)			

DASH, the Disabilities of the Arm, Shoulder and Hand.

## Data Availability

The data that support the findings of this study are available on request from the corresponding author.
